# Draft Genome Sequence of Leptospira interrogans Serovar Bataviae Strain D64, Isolated from the Urine of an Asymptomatic Dog in Pathum Thani, Thailand

**DOI:** 10.1128/MRA.00361-20

**Published:** 2020-07-23

**Authors:** Pannawich Boonciew, Alongkorn Kurilung, Kerstin Altheimer, Katrin Hartmann, Nuvee Prapasarakul

**Affiliations:** a Department of Veterinary Microbiology, Faculty of Veterinary Science, Chulalongkorn University, Bangkok, Thailand; b Clinic of Small Animal Internal Medicine, Centre of Clinical Veterinary Medicine, LMU Munich, Munich, Germany; c Diagnosis and Monitoring of Animal Pathogens Research Unit, Faculty of Veterinary Science, Chulalongkorn University, Bangkok, Thailand; University of Delaware

## Abstract

Leptospira interrogans serovar Bataviae is one of the serovars that can infect dogs. We report the draft genome sequence of Leptospira interrogans serovar Bataviae strain D64, which was isolated from the urine of an asymptomatic dog in Pathum Thani, Thailand, in 2017.

## ANNOUNCEMENT

Leptospirosis is an important infectious zoonotic disease caused by infection with pathogenic serovars of *Leptospira* (1). The disease occurs worldwide, particularly in tropical and subtropical regions, including Thailand (2). Leptospirosis is considered a significant health problem for humans, who are infected through mammals, mainly rodents, dogs, and cattle. Animals play an essential role through the maintenance of *Leptospira* spp. in their kidneys, shedding them into the environment via their urine (3, 4). In Bangkok, Thailand, and metropolitan areas, the seroprevalence in stray dogs was observed to be 12.1 to 83.5%, and *Leptospira interrogans* serovar Bataviae was predominant (5, 6). In this study, we present the draft genome sequence of an L. interrogans strain that was isolated from the urine of an asymptomatic dog in Pathum Thani, Thailand, in 2017.

Leptospira interrogans serovar Bataviae strain D64 was isolated from dog urine and was identified by urine culture, real-time PCR, and phylogenetic analysis, as described previously (5). Strain D64 was cultured at 28°C for 14 to 28 days in *Leptospira* medium base Ellinghausen-McCullough-Johnson-Harris (EMJH) (Thermo Fisher Scientific, USA) (7) supplemented with *Leptospira* enrichment EMJH (Thermo Fisher Scientific) and 3% rabbit serum (Thermo Fisher Scientific) under aerobic conditions and was observed by dark-field microscopy. The DNA was extracted with the DNeasy blood and tissue kit (Qiagen, Germany). The library was prepared and sequenced with the Nextera DNA Flex library preparation kit and the NovaSeq 6000 system with 150-bp paired-end run cycles (Illumina, USA). The genome reads were quality checked using FastQC v.0.11.8 (8). The genome assembly was carried out using A5-miseq v.20160825 (9). The genome statistics were evaluated using QUAST v.4.4 (10). The genome completeness was estimated using CheckM v.1.0.18 (11). The genome sequence was annotated with the NCBI Prokaryotic Genome Annotation Pipeline (PGAP) (12). All software used default parameters. For phylogenetic analysis, the strain D64 sequence was compared to the genomes in GenBank with the BLASTn algorithm using online NCBI BLAST v.2.10.1 with default parameters (https://blast.ncbi.nlm.nih.gov/Blast.cgi) (13, 14).

After assembly processing, the whole-genome sequence of strain D64 yielded a total of 81 contigs and 77 scaffolds, which covered a total of 4,773,473 bp with 10,590,394 paired-end reads, an *N*_50_ value of 160,740 bp, and an average coverage of 320×. The completeness of the genome was estimated to be 96.47%. The G+C content was estimated to be 35.1%. The annotated genome sequence was predicted to contain a total of 4,043 coding sequences, with 37 tRNA genes and 3 rRNA genes. The whole-genome sequence comparison of strain D64 revealed 98.9% identity with the sequence for Leptospira interrogans serovar Bataviae strain Kariadi-Satu in the NCBI GenBank database (accession number AHQF00000000). Sequencing was performed to identify the sequence type (ST) by multilocus sequence typing (MLST) analysis with seven housekeeping genes of *Leptospira* using the public MLST online server (software v.2.0.4) (https://cge.cbs.dtu.dk/services/MLST) of the Center for Genomic Epidemiology with default parameters (15). MLST analysis identified seven housekeeping genes (*caiB*, *glmU*, *mreA*, *pfkB*, *pntA*, *sucA*, and *tpiA*) of strain D64. The MLST profile of this strain was ST50. This genome information will provide insight into the epidemiology of the Thai L. interrogans serovar Bataviae strain and support disease control strategies. The pangenome (resistome, virulome, adaptation, and evolution) in the carrier dog will be studied further.

### Data availability.

The whole-genome sequence for Leptospira interrogans serovar Bataviae strain D64 was deposited in DDBJ/ENA/GenBank under the accession number WUMI00000000. The raw sequence reads were deposited in the NCBI Sequence Read Archive (SRA) under the BioProject accession number PRJNA597667.
